# Anti-GBM/ANCA double-positive crescentic nephritis in an elderly patient presenting with heart failure-like symptoms: A case report

**DOI:** 10.1097/MD.0000000000051225

**Published:** 2026-10-23

**Authors:** Meijuan Shi, Guoqing Shi, Hongmei Liu, Yao Huang, Nan Luo, Yumei Ge, Jie Zhu

**Affiliations:** a Department of Clinical Laboratory, Zhejiang Provincial People’s Hospital Bijie Hospital (The First People’s Hospital of Bijie), Bijie, Guizhou, China; b Department of Nephrology and Rheumatology, Zhejiang Provincial People’s Hospital Bijie Hospital (The First People’s Hospital of Bijie), Bijie, Guizhou, China.

**Keywords:** anca-associated vasculitis, anti-glomerular basement membrane disease, case reports, double-positive, rapidly progressive glomerulonephritis

## Abstract

**Rationale:**

Anti-glomerular basement membrane (anti-GBM) antibody disease and antineutrophil cytoplasmic antibody (ANCA)-associated vasculitis represent the 2 leading causes of rapidly progressive glomerulonephritis (RPGN). The coexistence of these conditions is rare, typically progresses rapidly, and has a poor prognosis. Elderly patients often present with nonspecific systemic symptoms, increasing the likelihood of misdiagnosis and treatment delay.

**Patient concerns:**

A 74-year-old woman presented with a one-month history of intermittent shortness of breath, worsening progressively over ten days, accompanied by nausea and vomiting. She denied hemoptysis, gross hematuria, rash, or arthralgia.

**Diagnoses:**

Initial assessment suggested heart failure. However, laboratory results revealed severe renal impairment (creatinine: 1025.99 μmol/L). Renal biopsy demonstrated crescent formation in over 80% of glomeruli, with immunofluorescence showing linear immunoglobulin G deposits along glomerular capillary loops. Serological testing revealed markedly elevated anti-GBM antibody (>400 RU/mL) and MPO-ANCA (108.4 RU/mL) levels, together with a perinuclear ANCA (P-ANCA) pattern on indirect immunofluorescence, supporting the diagnosis of anti-GBM glomerulonephritis with concomitant MPO-ANCA positivity.

**Interventions:**

Treatment included methylprednisolone pulse therapy, cyclophosphamide, and 3 plasma exchange sessions. Additional supportive measures comprised hemodialysis, antihypertensive therapy, and management of renal anemia.

**Outcomes:**

Although the patient’s uremic symptoms improved markedly, renal function did not recover. She was discharged on hemodialysis and was subsequently transitioned to maintenance peritoneal dialysis. By January 2026, the anti-GBM antibody level had decreased markedly to 4.27 RU/mL, below the positive cutoff, and MPO-ANCA had also become negative. At the last follow-up in April 2026, the patient remained dialysis-dependent and experienced recurrent hospitalizations for pneumonia, heart failure, and acute pancreatitis.

**Lessons:**

This case emphasizes that elderly patients with anti-GBM/ANCA double-positive crescentic glomerulonephritis may initially present with nonspecific symptoms, increasing the risk of incorrect diagnosis. Although combination therapy resulted in symptomatic improvement and serological remission, renal function failed to recover and dialysis dependence persisted. Screening for anti-GBM antibody and ANCA should therefore be considered in patients with unexplained rapidly progressive deterioration of kidney function or active urinary sediment, and renal biopsy should be performed promptly when clinically indicated.

## 1. Introduction

Rapidly progressive glomerulonephritis (RPGN) is a group of syndromes characterized by hematuria, proteinuria, and rapid deterioration of renal function. RPGN is the most severe form of glomerulonephritis, typically presenting as crescentic nephritis.^[1]^ Anti-glomerular basement membrane (anti-GBM) disease and antineutrophil cytoplasmic antibody (ANCA)-associated vasculitis (AAV) are the 2 primary autoimmune diseases causing RPGN.^[2]^ The coexistence of both antibodies in a patient, termed “double-positive nephritis,” accounts for approximately 20 to 40% of anti-GBM disease cases.^[3]^ Such patients typically present with more severe renal impairment and a higher risk of end-stage renal disease.^[4]^ A systematic review involving 538 double-positive patients reported a 1-year renal survival rate of only 38.7%, with myeloperoxidase antineutrophil cytoplasmic antibody (MPO-ANCA) being the predominant ANCA subtype (72.1%).^[5]^ Currently, its pathogenesis remains unclear, though the “epitope spreading” hypothesis is one of the prevailing theories.^[6]^ This article reports an elderly patient with double-positive crescentic nephritis initially presenting as heart failure. We aim to enhance clinical understanding and guide practice through an in-depth analysis of this atypical clinical manifestation.

## 2. Case presentation

A 74-year-old female patient was admitted to the hospital on August 17, 2025, due to “intermittent shortness of breath for one month, worsened with nausea and vomiting for ten days.” Symptoms were activity-related, without hemoptysis, hematuria, rash, or arthralgia. The patient had a history of hypertension but did not receive regular treatment. Due to these symptoms, she was initially diagnosed with “cardiac insufficiency” and admitted to the Department of Cardiovascular Medicine.

On admission, the patient had a temperature of 36.7°C, blood pressure of 167/82 mm Hg, and a heart rate of 50 beats/minutes. Physical examination showed pale eyelids and nail beds, clear breath sounds without rales in both lungs, and no edema in the lower limbs. Cardiac insufficiency was initially suspected based on her symptoms; however, objective findings did not support primary acute cardiac dysfunction. The NT-proBNP level was 2125.85 ng/L, echocardiography showed preserved left ventricular systolic function with a left ventricular ejection fraction of 56%, and chest CT showed no evidence of acute pulmonary edema. In addition, physical examination revealed no jugular venous distension, lower-extremity edema, or pulmonary rales. These findings suggested that her dyspnea was more likely multifactorial rather than due to primary cardiac dysfunction. Later in the disease course, the patient developed overt heart failure, with the left ventricular ejection fraction decreasing to 43%.

Laboratory tests revealed urinary protein (3+), urinary hemoglobin (3+), 24-hour urinary protein (0.76 grams/24 hours), serum creatinine (1025.99 μmol/L), blood urea nitrogen (32.87 mmol/L), and hemoglobin (96 g/L) (Table 1). The patient was subsequently referred to the Department of Nephrology and Rheumatology.

**Table 1:** Laboratory tests.

Component	Value	Reference range	Interpretation
Urinalysis			
Urinary protein	3+	Negative	Increased
Red blood cells (cells/HPF)	449	0–7	Increased
White blood cells (cells/HPF)	24	0–14	Increased
24-h urinary protein excretion (g/24 h)	0.76	0–0.15	Increased
Urinary glucose	Negative	Negative	Normal
Peripheral blood analysis			
White blood cells (×10^9^/L)	5.37	3.5–9.5	Normal
Neutrophils (×10^9^/L)	4.05	1.8–6.3	Normal
Red blood cells (×10^12^/L)	3.39	3.8–5.1	Decreased
Hemoglobin (g/L)	96	115–150	Decreased
Platelets (×10^9^/L)	157	125–350	Normal
Serum chemistry			
Serum creatinine (μmol/L)	1025.99	41–81	Increased
Blood urea nitrogen (mmol/L)	32.87	3.1–8.8	Increased
Uric acid (μmol/L)	682.80	155–357	Increased
eGFR (mL/min/1.73 m^2^)	4.76	≥90	Decreased
Total protein (g/L)	75.61	65–85	Normal
Serum albumin (g/L)	40.10	40–55	Normal
Aspartate aminotransferase (U/L)	14.00	13–35	Normal
Alanine aminotransferase (U/L)	9.00	7–40	Normal
Potassium (mmol/L)	4.47	3.5–5.3	Normal
Sodium (mmol/L)	140.40	137–147	Normal
Calcium (mmol/L)	2.31	2.2–2.7	Normal
Phosphorus (mmol/L)	2.27	0.85–1.51	Increased

eGFR = estimated glomerular filtration rate.

Imaging examinations indicated pulmonary emphysema in the upper lobes and minor inflammation in the lower lobes of both lungs. Renal ultrasound showed normal kidney sizes (right kidney 10.5 cm, left kidney 10.2 cm), slightly enhanced cortical echoes, and a clear cortico-medullary boundary, not indicative of chronic renal atrophy.

Renal biopsy: Immunofluorescence microscopy demonstrated strong linear immunoglobulin G deposition (3+) along the glomerular capillary loops, a pathognomonic feature of anti-GBM disease. Diffuse global C3 deposition (2+) was observed along the capillary loops and within the mesangial areas, suggesting activation of the complement pathway. Immunoglobulin A (IgA) staining was weak (1+) and showed a comma-shaped pattern of mesangial deposition. Because IgA was neither dominant nor codominant relative to immunoglobulin G, concomitant IgA nephropathy could not be confirmed. Albumin staining was negative in the glomeruli (Fig. 1). Electron microscopy showed segmental thinning of the GBM, extensive fusion of foot processes (>95%), proliferation of mesangial cells, increased mesangial matrix, and small amounts of electron-dense material in some basement membranes and mesangial regions (Fig. 2). Light microscopy identified 15 glomeruli: 3 with global glomerulosclerosis (20%), 3 cellular crescents, 7 fibrous crescents, and 2 fibrocellular crescents, totaling 80% crescent formation (12 of 15 glomeruli), consistent with crescentic glomerulonephritis. No overt fibrinoid necrosis of the glomerular capillary loops or arterioles was observed, and no normal glomeruli were preserved. Extensive interstitial fibrosis and tubular atrophy involved approximately 40 to 50% of the renal cortex, accompanied by diffuse interstitial inflammatory cell infiltration (Fig. 3).

**Figure 1.**
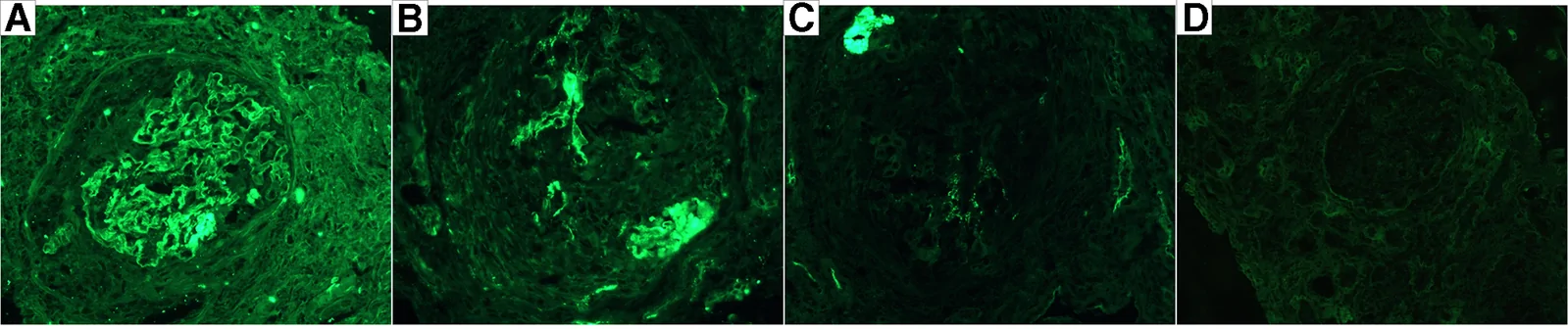
Immunofluorescence microscopy of renal biopsy. Immunofluorescence demonstrated linear deposition of IgG (3+) along the glomerular capillary loops, diffuse global deposition of C3 (2+) along the capillary loops and within the mesangial areas, comma-shaped deposition of IgA (1+) within the mesangium, and absence of ALB deposition in the glomeruli. (A): IgG; (B): C3; (C): IgA; (D): ALB. ALB = albumin, IgA = immunoglobulin A, IgG = immunoglobulin G.

**Figure 2.**
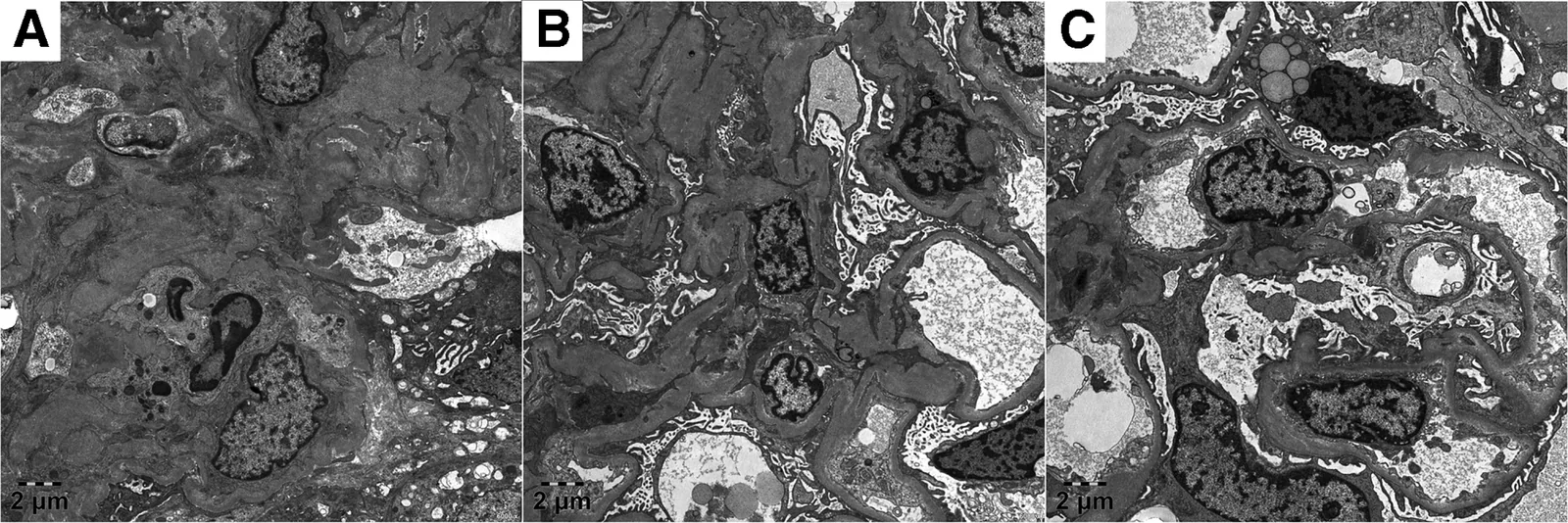
Transmission electron microscopy of renal biopsy. (A) Segmental thinning and wrinkling of the GBM, with extensive fusion of visceral epithelial cell foot processes. (B) Widening of the mesangial areas with increased mesangial cells and matrix. (C) Scattered small electron-dense deposits within portions of the basement membranes and mesangial regions. Scale bar = 2 μm. GBM = glomerular basement membrane.

**Figure 3.**
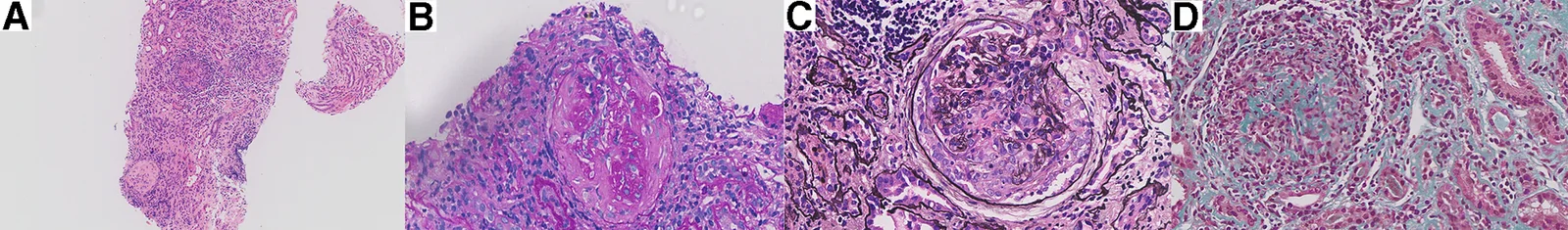
Light microscopy of renal biopsy. (A) Hematoxylin and eosin (HE) staining illustrating tissue structure and cellular morphology (×100). (B) Periodic acid-Schiff (PAS) staining highlighting the basement membrane and mesangial area of glomerular capillary loops (×400). (C) Periodic acid-silver methenamine (PASM) staining showing details of the GBM and mesangial region (×400). (D) Masson trichrome staining demonstrating collagen deposition and fibrosis (×400). GBM = glomerular basement membrane.

Immunoserological testing revealed an anti-GBM antibody level > 400.00 RU/mL, an MPO-ANCA level of 108.4 RU/mL, and P-ANCA positivity by indirect immunofluorescence. ANA and anti-dsDNA antibody tests were negative (Table 2). All antibody tests were performed before plasma exchange.

**Table 2:** Immunoserologic tests.

Component	Method	Result	Reference range	Interpretation
Anti-GBM antibodies	Chemiluminescence immunoassay	>400 RU/mL	<20 RU/mL	Increased
MPO-ANCA	Chemiluminescence immunoassay	108.4 RU/mL	<20 RU/mL	Increased
PR3-ANCA	Chemiluminescence immunoassay	<2.00 RU/mL	<20 RU/mL	Normal
P-ANCA	Indirect immunofluorescence	Positive	Negative	Positive
C-ANCA	Indirect immunofluorescence	Negative	Negative	Normal
ANA	Chemiluminescence immunoassay	4.52 RU/mL	<20 RU/mL	Normal
dsDNA	Chemiluminescence immunoassay	2.46 IU/mL	<20 IU/mL	Normal

ANA = antinuclear antibody, anti-GBM = anti-glomerular basement membrane antibody, C-ANCA = cytoplasmic antineutrophil cytoplasmic antibody, dsDNA = double-stranded DNA, MPO-ANCA = myeloperoxidase antineutrophil cytoplasmic antibody, P-ANCA = perinuclear antineutrophil cytoplasmic antibody, PR3-ANCA = proteinase 3 antineutrophil cytoplasmic antibody.

## 3. Treatment and outcome

According to the pathology and immunoserological results, the patient was diagnosed with anti-GBM glomerulonephritis with concomitant MPO-ANCA positivity. After diagnosis, plasma exchange-based immunosuppressive therapy was initiated promptly. Three consecutive daily plasma exchange sessions were performed from August 27–29, 2025, with 1600 mL of group O, Rh(D)-positive fresh frozen plasma exchanged during each session. The patient’s blood type was confirmed as group O, Rh(D)-positive, ensuring ABO compatibility of the replacement fluid. This volume was determined according to the patient’s estimated plasma volume (approximately 40 mL/kg body weight). At admission, the patient had a body weight of 67 kg and an estimated total plasma volume of approximately 2680 mL, with each plasma exchange accounting for roughly 60% of the estimated plasma volume. Plasma exchange was discontinued after completion of the planned 3 sessions. We note that the single exchange volume was lower than the generally recommended dose of 1.0 to 1.5 plasma volumes per session. The decision to reduce the dose and to discontinue plasma exchange after 3 induction sessions was based on the treating physician’s clinical assessment, which took into account the patient’s advanced age, dialysis-dependent severe kidney failure, and anemia. However, for anti-GBM disease, the standard course of plasma exchange is typically continued until antibody clearance is achieved, and the shorter course in this case should be considered a potential limitation. The patient also received intravenous methylprednisolone at 500 mg/day for 3 days, followed by oral prednisone at 40 mg/day, which was gradually tapered, reaching 10 mg/day on March 10, 2026. Because the patient was also MPO-ANCA positive, low-dose prednisone was continued as maintenance therapy. The patient received 8 intravenous cyclophosphamide pulses: 0.6 grams on August 27, 2025; 0.4 grams on August 28, 2025; 0.4 grams on September 27, 2025; 0.6 grams on September 28, 2025; 0.4 grams on November 30, 2025; 0.4 grams on December 1, 2025; 0.4 grams on January 3, 2026; and 0.2 grams on March 1, 2026, for a cumulative dose of 3.4 grams. The timing and dosage of cyclophosphamide were adjusted during treatment according to the presence of infection, blood cell counts, and the patient’s overall clinical status. Treatment was generally well tolerated; however, nausea, vomiting, and abdominal distension developed after the cyclophosphamide pulses administered on September 27–28, 2025. Cyclophosphamide was therefore temporarily withheld, and the gastrointestinal symptoms improved after antiemetic, gastroprotective, and prokinetic treatment. During the episode of pneumonia in October–November 2025, subsequent cyclophosphamide administration was postponed until the infection had improved. When pneumonia recurred in February–March 2026, the final cyclophosphamide dose was reduced to 0.2 grams on March 1, 2026. Following the diagnosis of acute pancreatitis later in March 2026, no further cyclophosphamide was administered. Prednisone was reduced to 10 mg/day on March 10 and was temporarily discontinued on March 30 because of poor oral intake. Throughout follow-up, anti-GBM antibody levels, MPO-ANCA, routine urinalysis, serum creatinine, and complete blood counts were monitored regularly. Prophylactic trimethoprim-sulfamethoxazole was administered for prevention of Pneumocystis jirovecii pneumonia because of the intensive immunosuppressive regimen. Hemodialysis was initiated due to oliguria with a 24-hour urine output of approximately 400 to 500 mL, severe uremic symptoms including nausea, vomiting, and fatigue, and a markedly elevated serum creatinine level of 1025.99 μmol/L. Antihypertensive therapy, erythropoietin, iron supplementation, and other supportive treatments were also provided.

After treatment, the patient’s gastrointestinal symptoms and dyspnea resolved, and appetite improved. However, renal function did not recover. The absence of renal recovery was best explained by the severe chronic changes on renal biopsy, including the absence of normal glomeruli, 40 to 50% interstitial fibrosis with tubular atrophy, and predominantly fibrous and fibrocellular crescents. The requirement for dialysis at diagnosis further indicated a poor renal prognosis. Serum creatinine remained approximately 600 μmol/L, and ongoing hemodialysis was required at discharge on September 1, 2025.

At the 7.5-month follow-up in April 2026, the patient remained on maintenance peritoneal dialysis after transitioning from hemodialysis, with serum creatinine levels ranging from 600 to 860 μmol/L. By January 2026, the anti-GBM antibody level had decreased markedly to 4.27 RU/mL, below the positive cutoff, and MPO-ANCA had also become negative. Nevertheless, the patient experienced recurrent hospitalizations for newly developed heart failure, pneumonia, and acute pancreatitis. The pneumonia and acute pancreatitis occurred during the immunosuppressive phase, their causality with immunosuppressive therapy remains uncertain.

The patient’s clinical course from diagnosis through follow-up is summarized in Figure 4.

**Figure 4.**
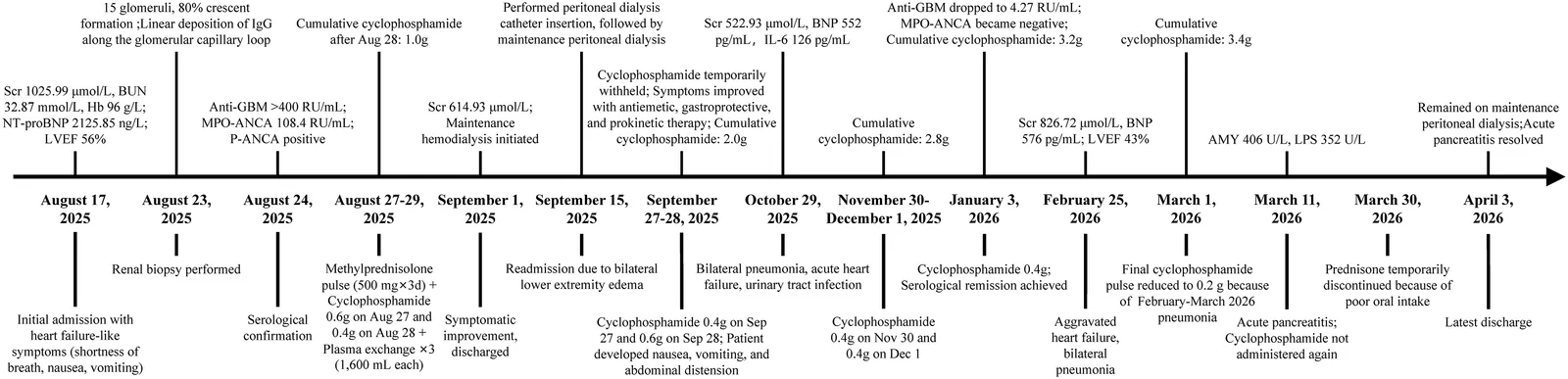
Clinical timeline of the patient’s disease course.

## 4. Discussion

Both anti-GBM and ANCA antibodies can cause crescentic glomerulonephritis and rapid deterioration of renal function. The clinical presentation can simultaneously include clinical, pathological, and prognostic characteristics of anti-GBM disease and AAV.^[7,8]^ The coexistence of these antibodies in the same patient raises important questions regarding the underlying pathogenesis. One widely proposed hypothesis, although it cannot be definitively established on the basis of a single antibody assessment, is the “epitope spreading” model.^[6,8]^ According to this hypothesis, subclinical MPO-AAV may initially cause inflammatory injury to the GBM, resulting in exposure of normally sequestered antigens, such as the Goodpasture epitope of the α3 chain of type IV collagen. These exposed antigens are then recognized by the immune system, triggering an autoimmune response against GBM and production of high-titer anti-GBM antibodies. Ultimately, the 2 antibodies act synergistically, causing a more aggressive immune attack on the kidneys. In this case, the MPO-ANCA level (108.4 RU/mL) was numerically lower than the anti-GBM antibody level (>400 RU/mL). However, these measurements were obtained using different chemiluminescence immunoassay systems with distinct calibrators and therefore cannot be directly compared. The apparent predominance of the anti-GBM antibody response should thus be interpreted with caution, and any inference regarding the temporal sequence of antibody development remains speculative.

The core clinical implication of this case is its atypical initial presentation. The 74-year-old patient presented with intermittent shortness of breath, nausea, and vomiting, without typical features such as gross hematuria, hemoptysis, rash, or arthralgia. Physical examination upon admission revealed no typical signs of pulmonary edema. CT imaging also showed no acute pulmonary edema, only chronic emphysema and mild inflammation, initially suggesting a cardiovascular diagnosis. The manifestations of kidney and lung involvement differed from previous reports of double-positive nephritis.^[9]^ The patient’s shortness of breath was likely caused by a combination of severe renal failure and renal anemia. This case expands the clinical spectrum of double-positive nephritis in the elderly. Patients with double-positive nephritis have a high incidence in middle-aged and elderly populations.^[10]^ Elderly patients often have multiple underlying conditions and nonspecific systemic symptoms, increasing the likelihood of masking renal lesions and the risk of misdiagnosis.^[11]^ Clinicians should include rapidly progressive nephritis in the differential diagnosis when elderly patients present with unexplained “heart failure-like symptoms” or acute general discomfort. Serum creatinine measurement, urinalysis, and screening for anti-GBM antibodies and ANCA should be performed promptly in such patients.

At the treatment level, rapid immune intervention combined with targeted antibody clearance is central to treating double-positive crescentic glomerulonephritis.^[12]^ In this case, the regimen of plasma exchange, methylprednisolone pulse, and cyclophosphamide immunosuppression was adopted. Supplementary supportive treatments, including hemodialysis, antihypertensive therapy, and correction of renal anemia, effectively relieved systemic symptoms and controlled disease activity. More than one month elapsed from symptom onset to diagnosis in this patient. Renal biopsy revealed crescents in 80% of glomeruli, predominantly cellular-fibrous and fibrous types, indicating that lesions had entered the chronic stage.^[13]^ Previous reports suggest that initial serum creatinine > 600 μmol/L or dialysis dependence strongly predicts poor renal prognosis.^[14,15]^ The outcome of this case aligns with these findings, highlighting the crucial importance of early diagnosis. Compared to patients with anti-GBM disease alone, although the proportion of complete renal recovery after treatment remains low, patients with double-positive nephritis may have relatively better survival outcomes.^[8]^

Several limitations of this case report should be acknowledged. First, as a single case report, the findings may not be generalizable to the broader population of patients with double-positive crescentic nephritis. Second, because serial antibody measurements before clinical onset were unavailable, the temporal relationship between MPO-ANCA and anti-GBM antibody development could not be definitively determined. Third, although the patient achieved serological remission, with the anti-GBM antibody level falling below the positive cutoff and MPO-ANCA becoming negative, the follow-up period was limited to 7.5 months. Longer-term follow-up is therefore required to fully evaluate renal, cardiovascular, and overall clinical outcomes.

## 5. Conclusion

In conclusion, we report an elderly patient with anti-GBM/ANCA double-positive crescentic glomerulonephritis who initially presented with heart failure-like symptoms. Following treatment with plasma exchange, methylprednisolone pulse therapy, and cyclophosphamide, the patient’s gastrointestinal symptoms and dyspnea improved, and serological remission was achieved. However, renal function did not recover, and the patient remained dialysis-dependent. The nonspecific initial presentation in this case illustrates the diagnostic challenges posed by double-positive disease in elderly patients. In clinical practice, anti-GBM antibody and ANCA screening should be considered in patients with unexplained rapidly progressive kidney dysfunction or active urinary sediment, and renal biopsy should be performed without delay when clinically indicated. The mechanism of “epitope spreading” merits further basic research.

## Acknowledgments

We gratefully acknowledge the patients and pathologists for their contributions to this study.

## Author contributions

**Conceptualization:** Yao Huang, Yumei Ge.

**Data curation:** Hongmei Liu.

**Investigation:** Yao Huang, Nan Luo.

**Resources:** Guoqing Shi, Nan Luo, Yumei Ge.

**Writing – original draft:** Meijuan Shi.

**Writing – review & editing:** Jie Zhu.

## References

[1] Chaudhury GN Khandoker T Ferdous T Chowdhury RA Afroz S Hanif M. Clinicopathological spectrum and outcome of rapidly progressive glomerulonephritis. Asian J Pediatr Nephrol. 2020;3:78–81.

[2] Ivković V Bajema I Bruchfeld A. Efficacy and safety of rituximab in antiglomerular basement membrane disease. Kidney Int Rep. 2025;10:743–52. 10.1016/j.ekir.2024.12.026 PMC11993202 40225363

[3] Kunaprayoon L Scheffel ETC Abdel-Rahman EM. Management of double-seropositive anti-glomerular basement membrane and anti-neutrophil cytoplasmic antibodies with 100% crescentic glomerulonephritis and nephrotic range proteinuria in a young female. Biomedicines. 2024;12:906. 10.3390/biomedicines12040906 PMC11048036 38672260

[4] Hu XL Shen CJ Meng T. Clinical features and prognosis of MPO-ANCA and anti-GBM double-seropositive patients. Front Immunol. 2022;13:991469. 10.3389/fimmu.2022.991469 PMC9648717 36389826

[5] Philip R Dumont A Martin Silva N de Boysson H Aouba A Deshayes S. ANCA and anti-glomerular basement membrane double-positive patients: a systematic review of the literature. Autoimmun Rev. 2021;20:102885. 10.1016/j.autrev.2021.102885 34242834

[6] Strizzi CT Ambrogio M Zanoni F. Epitope spreading in immune-mediated glomerulonephritis: the expanding target. Int J Mol Sci . 2024;25:11096. 10.3390/ijms252011096 PMC11507388 39456878

[7] Tang AQ Zhao X Tao T. Unleashing the power of complement activation: unraveling renal damage in human anti-glomerular basement membrane disease. Front Immunol. 2023;14:1229806. 10.3389/fimmu.2023.1229806 PMC10540768 37781380

[8] McAdoo SP Tanna A Hrušková Z. Patients double-seropositive for ANCA and anti-GBM antibodies have varied renal survival, frequency of relapse, and outcomes compared to single-seropositive patients. Kidney Int. 2017;92:693–702. 10.1016/j.kint.2017.03.014 PMC5567410 28506760

[9] Koirala A Sharma PD Jhaveri KD Jain K Geetha D. Rapidly progressive glomerulonephritis. Adv Kidney Dis Health. 2024;31:485–95. 10.1053/j.akdh.2024.08.006 39577882

[10] Mohamed ON Ibrahim SA Saleh RK. Clinicopathological characteristics and predictors of outcome of rapidly progressive glomerulonephritis: a retrospective study. BMC Nephrol. 2024;25:103. 10.1186/s12882-024-03532-y PMC10949592 38500101

[11] Aucella F Corsonello A Leosco D Brunori G Gesualdo L Antonelli-Incalzi R. Beyond chronic kidney disease: the diagnosis of renal disease in the elderly as an unmet need. A position paper endorsed by Italian Society of Nephrology (SIN) and Italian Society of Geriatrics and Gerontology (SIGG). J Nephrol. 2019;32:165–76. 10.1007/s40620-019-00584-4 PMC6423311 30659521

[12] Omotoso BA Cathro HP Balogun RA. Crescentic glomerulonephritis with dual positive anti-GBM and C-ANCA/PR3 antibodies. Clin Nephrol Case Stud. 2016;4:5–10. 10.5414/CNCS108666 PMC5438001 29043135

[13] Kuang H Lin CX Yu XJ Cui Z Zhao MH Jia XY. Bowman’s capsule rupture and its clinical significance in patients with anti-glomerular basement membrane disease. Front Immunol. 2025;16:1655319. 10.3389/fimmu.2025.1655319 PMC12711854 41425590

[14] van Daalen EE Jennette JC McAdoo SP. Predicting outcome in patients with anti-GBM glomerulonephritis. Clin J Am Soc Nephrol. 2018;13:63–72. 10.2215/CJN.04290417 PMC5753308 29162595

[15] Levy JB Hammad T Coulthart A Dougan T Pusey CD. Clinical features and outcome of patients with both ANCA and anti-GBM antibodies. Kidney Int. 2004;66:1535–40. 10.1111/j.1523-1755.2004.00917.x 15458448

